# Moving Advertisements Systematically Affect Gaze Behavior and Performance in the Soccer Penalty Kick

**DOI:** 10.3389/fspor.2019.00069

**Published:** 2020-01-14

**Authors:** Gareth Paterson, John van der Kamp, Geert Savelsbergh

**Affiliations:** ^1^Amsterdam Movement Sciences, Faculty of Behavioural and Movement Sciences, Vrije Universiteit Amsterdam, Amsterdam, Netherlands; ^2^Research Centre for Exercise, School and Sport, Windesheim University of Applied Sciences, Zwolle, Netherlands; ^3^Academy for Physical Education, Amsterdam University of Applied Sciences, Amsterdam, Netherlands; ^4^Faculty of Sports and Nutrition, Amsterdam University of Applied Sciences, Amsterdam, Netherlands

**Keywords:** penalty kick, far aiming, visual search, visual attention, duncker illusion

## Abstract

The aim of the current study was to investigate whether a moving advertisement positioned behind the goal area would influence the visual attention of participants performing a soccer penalty kick, and, whether this would an effect on subsequent motor performance. It was hypothesized that if the (moving) advertisement would function as a distractor, then this would result in non-specific disruptions in penalty performance measures, especially affecting aiming location and precision. Alternatively, it was reasoned that, in line with the Dunker illusion, the moving advertisement would systematically affect perception of target location, resulting in changes in penalty performance and aiming that are specific for the direction of motion of the advertisement. To test these hypotheses, we investigated the gaze behavior and kicking performance of intermediate skilled soccer players taking penalty kicks in three differing advertisement conditions, namely no advertisement, a stationary advertisement, and a moving advertisement. The latter condition consisted of an advertisement moving from left to right and an advertisement moving from right to left. Results showed that a moving advertisement placed behind the goal area indeed caught the visual attention of soccer penalty kickers using a goalkeeper-dependent kicking strategy. Participants kicking performance tended to be less variable within the no advertisement condition compared to the moving advertisement condition. In addition, systematic, direction-specific effects on aiming were found when comparing conditions in which the advertisement moved in opposite directions. This pattern of findings indicate that the accuracy of the penalty kick is impacted by task-irrelevant contextual information.

## Introduction

In soccer, penalty kicks are decisive events that can decide the outcome of a match. The average number of goals scored by both teams is typically low during regulation time in soccer matches (i.e., 2.5; Bar-Eli et al., 2007), and as a consequence, the scoring opportunity that is provided by a penalty kick can decide the outcome of a match. In addition, during a decisive penalty shoot-out, the importance to the outcome of the match is even more obvious.

In the penalty kick, the ball is placed 11 m from the goal area, which measures 24 ft (7.32 m) wide by 8 ft (2.44 m) high, giving the kicker a target area of 18 m^2^ to aim at. Further to this, a kick is struck with a typical speed of 22–27 m/s, with the ball reaching the goal line in approximately 600 ms (Wood et al., 2015; van der Kamp et al., 2018). Due to constraints on the time that the goalkeeper requires to cover the entire goal area, the overwhelming advantage is in favor of the kicker (Wood and Wilson, 2010; Noël and Van der Kamp, 2012). It is therefore surprising that a large percentage of penalty kicks are not converted, with ~20–25% of the shots being missed or saved (Mcgarry and Franks, 2000; Jordet et al., 2007).

With this in mind, researchers have shown a significant interest in uncovering factors that effect accuracy and success in the penalty kick. Specifically, recent advances in mobile gaze registration systems have led to an increase in empirical studies that have attempted to explore gaze behavior and visual attention within the performance of the penalty kick (e.g., Wilson et al., 2009; Wood and Wilson, 2010, 2011; Piras and Vickers, 2011; Van der Kamp, 2011; Noël and Van der Kamp, 2012). Evidently, and similar to other far aiming tasks (e.g., Helsen et al., 2000; Rodrigues et al., 2002; Vickers and Williams, 2007; Land, 2009), these studies have demonstrated a functional coupling of gaze behavior and kicking, with the information made available from gaze fixations being pertinent in decision making, as well as maintaining effective performance (Behan and Wilson, 2008). That is, within the soccer penalty situation, the kicker has to deal with both a proximal and a distal target, i.e., the foot has to hit the ball (proximal target) as accurately as possible with an sufficient amount of force, and secondly, the kick has to accelerate the ball toward a target location within the goal that is outside of the goalkeeper's reach (distal target).

The kicker generally adopts one of two penalty kick strategies, which include to either attempt to anticipate the direction of the goalkeeper dive during the run-up to the ball and kick to the opposite side of the goal at the last moment (i.e., the keeper-dependent strategy); or, to use a more controlled approach and decide the direction of the kick without taking the goalkeeper's actions into account during the run-up phase of the kick (i.e., keeper-independent strategy) (Van der Kamp, 2006, see also Kuhn, 1988). The two penalty kick strategies have been shown to invoke distinct patterns of gaze, which are directly associated with the success of penalty kicks. For example, Noël and Van der Kamp (2012) showed that the distinct pattern of gaze in the case of the keeper independent strategy allowed for more optimal control of the kicking movements as compared to the gaze pattern elicited by the keeper dependent strategy. Gaze behavior within the goalkeeper independent strategy was associated with prolonged focus on the inside areas of the goal (distal target), shorter times fixating the goalkeeper, and longer fixation times toward the ball (proximal target), all of which resulted in kicks that were less centralized and gave the goalkeeper less opportunity to save the ball (Noël and Van der Kamp, 2012; see also Wilson et al., 2009; Van der Kamp, 2011; Wood et al., 2015; Kurz et al., 2018).

In the competitive environment, there are a number of distractors that can potentially influence typical gaze patterns of kickers. Such shifting in attention to task irrelevant cues has the potential to disrupt motor performance (Beilock and Carr, 2001; Wood and Wilson, 2010; Morris, 2012; Lidor et al., 2013). One of the more modern potential distractions to visual attention in soccer stadiums includes billboards that are used to advertise during competitive matches. These boards can typically be seen on the entire perimeter of the field, including being placed directly behind the goal area. These modern LED (light emitting diode) display boards allow for multiple advertisements to be scrolled across their screens for the duration of a match. Advertisements appear and re-appear in differing formats and typically also include images that move from left to right, or right to left in direction. It is clear that a moving advertisement behind the goal has the potential to catch visual attention during a penalty task and thus to disrupt the typical gaze patterns of the kicker, with potential performance implications.

These performance implications can be two-fold. First, the attentional shift could have a non-specific effect on performance, in which the mere presence of the advertisement and/or the motion of the advertisement would result in a generic disruption of kicking performance measures including increased inaccuracy and/or precision in aiming. In other words, the (moving) advertisement may function as a distractor (e.g., Beilock and Carr, 2001). On the other hand, the attentional shift could have a specific effect on performance, that is, influencing kicking accuracy in a systematic manner depending on the direction of the moving advertisement. This would be analogous to the Duncker illusion (Duncker, 1929). Duncker (1929) demonstrated that background motion can induce an illusory perception of motion of a stationary foreground object. This illusory perceived motion of the object is in the direction opposite to that of the background motion (Zivotofsky, 2004). Most critically, it has been shown that such background motion can have similar effects on action, particularly within aiming tasks. Brouwer et al. (2003; see also Soechting et al., 2001) reported that background motion from left to right and right to left resulted in systematic aiming errors to the left and right of the target, respectively. This is in line with later findings that far aiming tasks are impacted by allocentric or contextual information (Van der Kamp and Masters, 2008; Van der Kamp et al., 2009; Shim et al., 2014).

The present study examines the effects of a stationary and moving advertisement behind the goal area on penalty kick performance. To this end, we investigated the gaze behavior and kicking performance of intermediate skilled soccer players taking penalty kicks in three differing advertisement conditions, namely no advertisement, a stationary advertisement, and a moving advertisement. The later condition consisted of an advertisement moving from left to right and an advertisement moving from right to left. Participants were enticed to use a keeper-dependent strategy and give themselves the best opportunity to score a goal by taking the goalkeepers dive into consideration when deciding in which direction to kick the ball^1^. We were interested to see whether the moving advertisement did in fact catch the kicker's attention and affect gaze behavior, and if so, whether this would disrupt subsequent penalty kick performance. We hypothesized that if the advertisement would serve as a distractor, then the disruption, if any, would be non-specific that is, it would result in an overall decrease in kicking performance measures (e.g., aiming location and/or precision). Alternatively, if the moving background would have specific effects, then systematic changes in kicking performance measures (e.g., aiming location) would be dependent on the direction of motion of the advertisement.

## Materials and Methods

### Participants

Sixteen intermediately skilled soccer players volunteered to participate in the study (mean age = 26.3, SD = 2.8 years, one female). Fifteen of the participants were right-footed with one of the participants being left-footed. All 16 players played in the national amateur leagues of the Royal Dutch Football Association (KNVB). The experiment was approved by the local ethics committee and all participants signed a written informed consent form before the start of the experiment.

### Material and Equipment

**Figure 1:**
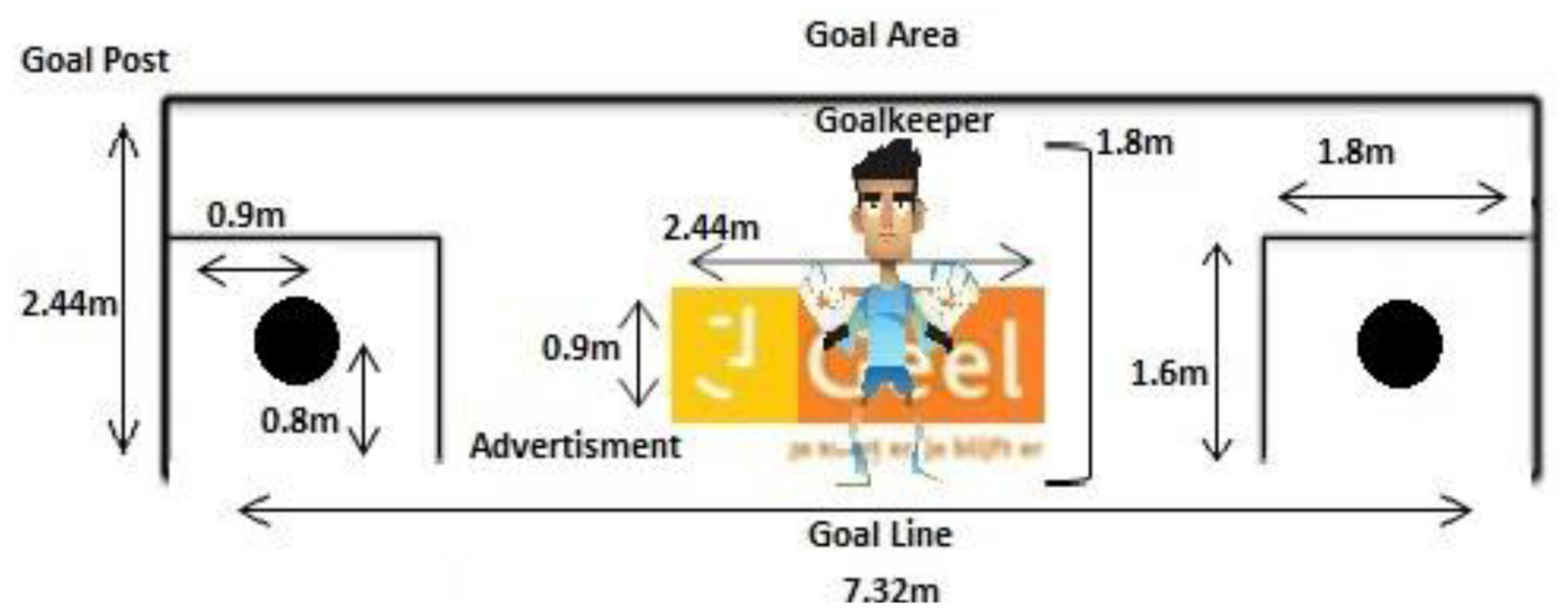
Frontal view of the experimental design, showing a standard size goal area with the two circular target area locations, the goalkeeper and the advertisement.

Eight different video clips were created showing a goalkeeper diving either to the left or right side, under three differing advertisement conditions. These video clips were recorded using a digital video camera (Kodak Playfull ZE2) from the perspective of a penalty kick taker. The advertising was projected onto a white wall (“goal area”) using a projector (Dell 1510X) with the goalkeeper standing in the middle of the goal area. The goalkeeper was instructed by the researcher to dive the left or the right side of the goal, under the following advertisement conditions: No advertising present/control (C), stationary advertisement (S), and moving advertisement (M), which (continuously) moved from either the left to the right (MLR), or from right to the left (MRL). The advertisement was a digital picture of 0.9 m in height, and 2.44 m in length made out of salient colors i.e., yellow and orange. The bottom of the advertisement was placed approximately 0.9 m from the ground when projected onto the screen (see Figure 1). This resulted in a total of eight clips, each 3.2 s in length. Windows Media Player editing software was used in order to synchronize the time between the start of the clip and goalkeeper movement across all eight clips. Accordingly, the clips showed the goalkeeper starting his movement at 1.8 s after the start of the clip. This allowed us to coordinate the goalkeeper movement to the participants' run-up phase (Figure 1).

**Figure 2:**
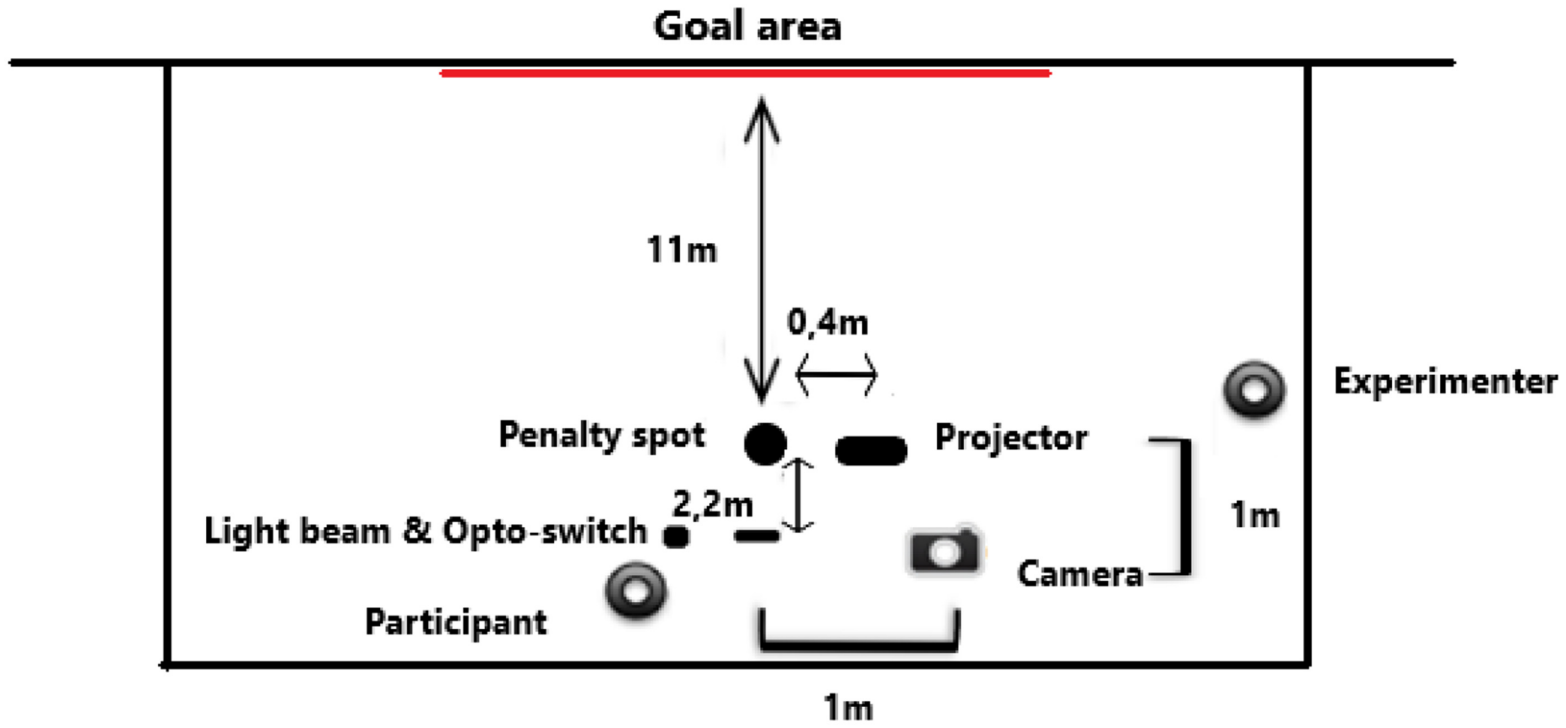
Birds-eye view of the experimental setup. The video clips were projected onto the goal area PVC canvas using a projector to the side of the penalty mark. The digital video camera was positioned to the side of the penalty mark, and the Opto-switch was positioned behind the penalty mark.

The experiment took place in an indoor sporting facility. The penalties were performed in accordance with official FIFA law, using a standard sized goal area (7.32 × 2.44 m), with the distance to the goal being 11 m from the penalty spot. A “FIFA-Approved” size 5 football with standard inflation was used. A white PVC canvas was attached to the goal (post and crossbar) (see Figure 2).

The video clips were projected onto the PVC canvas using a projector (Dell 1510X) that was located 40 cm to the side of the penalty mark. A digital video camera (Kodak Playfull ZE2) was positioned 1 m behind and 1 m to the side of the penalty mark in order to record the goal area. An Opto-switch (E3S-R 30E4 Omron) and light beam, positioned at knee height, were positioned 2.2 m behind the penalty mark. When participants walked through the switch, the beam was interrupted, and the clips were initiated (see Figure 2) and after 1.8 s, the goalkeeper initiated the dive. Based on pilot work, this was timed just before or at the moment of the participants' support foot landing next to the ball, but with trial-to-trial variability, depending on the participants' current run-up speed. A white background was projected onto the wall between the penalty kick trials.

Gaze behaviors were recorded using an Applied Science Laboratories (ASL; Bedford, MA) Mobile Eye-Tracker. The device measures eye-line of gaze at 25 Hz with respect to eye and scene cameras that are mounted on a pair of glasses, worn by the participant. The system records onto a modified DVCR, which was worn in a pouch around the waist of the participant. The DVCR is plugged into a laptop (Dell Inspiron 6400) with Eyevision recording software installed. A circular cursor, representing 1° of visual angle indicates the location of gaze on a video image of the scene (i.e., the system has an accuracy of ± 1° of visual angle and a precision of ±0.5°) was then recorded for offline analysis. The system was calibrated before each participant began the experiment by having participants look at nine specific target areas located on the PVC canvas representing the goal area. Participants stood on the penalty spot and were instructed to fixate on each of the pre-determined calibration points, one after the other. On completion of the calibration, participants were asked to view specific areas within the performance environment in order to verify the accuracy of the calibration. After each block of 12 penalties, the accuracy of calibration was checked with a recalibration only being performed in the case of line of gaze inaccuracy. A firewire cable was used to connect the DVCR to the laptop during calibration. Once calibration was complete, the firewire was removed, allowing the participant and eye tracker to be fully mobile. Data was saved onto the DVCR recorder and downloaded after the experiment in order to conduct offline analysis.

### Procedure and Design

After being fitted with the Mobile Eye and completing the calibration, participants were instructed to warm-up by performing 10 penalty shots at the blank PVC canvas, i.e., with no video clip being projected onto the canvas. During the 10 penalty warm up shots, the participants were required to aim for one of two target areas within the goal area i.e., left target and right target area. The two circular target area locations were 22 cm in diameter (similar to the diameter of the ball) and were black in color. The center of each target area was 0.8 m from the ground and 0.9 m from the goal post (see Figure 1).

After the warm-up was complete, the participants began the experiment with the following instructions. Participants were required to start their run-up at minimum distance of 3.5 m from behind the ball and were asked to take the penalty as they would in competition (using their preferred foot). Participants were told that when they interrupted the light beam, a video clip with a goalkeeper would project onto the goal area in front of them. The goalkeeper would dive to either the left, or right side of the goal after a short amount of time after appearing in the goal area. Participants were required to shoot the ball to the opposite side of the goal the goalkeeper was diving to. In order to be successful, the participants had to place the ball to the opposite side of the goalkeeper dive, toward the side of the goal area, as they would in competition, to give themselves the best chance of scoring a goal. The two circular target areas remained visible throughout the trial. The participants received no information about the advertisement that was projected onto the goal area within the differing advertisement conditions.

For each participant, the experiment started with the initial interruption of the light beam, which initiated the first video clip. In each of the advertisement conditions, the goalkeeper could dive to either to the left or right side of the goal area, with clips diving to the left or the right being completely randomized. A repeated measures design with differing conditions of 12 penalty kicks were used. The blocks were counterbalanced with the total of 48 penalty kicks (i.e., 12 each in the control, stationary, moving from left to right, and moving from right to left), lasting ~20 min per participant.

### Data Analysis

In a first round of analysis, we compared gaze and performance across the three advertisement conditions, namely C-, S-, and M-conditions to assess non-specific effects of the (moving) advertisement. In a second round, we focussed on systematic, direction-specific effects of the moving advertisement by comparing gaze and performance between the two moving MLR- and MRL-conditions. Finally, we also assessed non-specific and specific effects for only the trials that the participants looked to the moving advertisement between the MLR- and MRL-conditions.

#### Gaze Behavior

WIN-analyse software was used for a frame-by-frame analysis of the point of gaze (POG) recordings during the penalty trials, from the moment the participant initiated the run-up, until contact with the ball was made (total viewing time). Each frame was analyzed with each gaze fixation being divided into one of the following six areas of interest: Goalkeeper, ball, left target, right target, advertisement and “other.” The “other” area was every frame in which a participant did not look at either of the initial five areas of interest. After all trials were analyzed, gaze directed at each of the areas of interest was expressed as a percentage of total viewing time of the penalty kicks (see Van der Kamp, 2011). We also determined the percentage of trials during which participants were actually directing gaze toward the (moving) advertisement.

### Penalty Performance Measures

Video recordings were used to categorize each penalty kick as either a kick directed to the right or left of the goal, with an inter-reliability (*r* = 0.88, *p* < 0.05) and intra-reliability (*r* = 0.92, *p* < 0.05) of the observers, independent of the direction of the goalkeeper's dive. The penalty kicks were further categorized as either a score (i.e., a shot to the opposite side of the goalkeeper's dive, between the posts and crossbar), a save (i.e., a shot in the same direction of the goalkeeper dive, between the posts and the crossbar), or a miss (a shot that completely missed the goal area).

Subsequently, screenshots were created for each penalty kick at the moment of ball contact with the canvas (i.e., crossed the goal line) and with Kinovea Motion Analysis software, with the absolute distance in cm of the ball landing location from the center of the goal being determined to indicate the accuracy of aiming of the kick (i.e., absolute error). In addition, we took the standard deviation of the absolute distance in cm to determine the precision in aiming between conditions (i.e., variable error). Penalty kicks that completely missed the goal area were also included in the analysis with kicks outside of the video frame being assigned the maximal distance from the center of the goal to the edge of the video frame (i.e., 705 cm). Related to specific, directional effects on performance measures, we determined the signed distance in cm of the ball landing location from the goal center (i.e., constant error), with a negative value being allocated for locations to the left of the center of the goal, and a positive value being allocated for locations to the right of the center of the goal. The absolute and variable distance measures served as an indicator of distraction (i.e., aiming accuracy and precision), while the signed distance measure allowed the assessment of systematic, directional effects on penalty kick performance between the two moving MLR- and MRL-conditions.

### Statistics

The percentage viewing time to each of the areas of interest were analyzed with separate ANOVAs with repeated measures for the factor condition (i.e., C-, S-, M-conditions). It must be taken into account that the areas of interest are interdependent. When viewing time of one of the areas of interest increases, the sum of the viewing times of the other areas of interest must decrease, and vice versa. However, there does not exist a reciprocal relationship between two variables, and therefore we decided to report separate ANOVAs for the dependent variables (Kurz et al., 2018). A Huyn-Feldt correction to the degrees of freedom was applied in the case of any violations of sphericity and partial eta-squared ($\eta_{\text{p}}^{2}$) values were computed to determine the proportion of total variability attributable to each factor. *Post-hoc* pairwise comparisons were conducted using the Bonferroni correction procedure to identify where the specific differences occurred between the conditions. Subsequently, we used paired *t*-test to compare difference in percentage viewing time of each of the areas of interest for the MLR-condition and MRL-condition.

Similarly, for the penalty kick performance measures including the score, save and miss percentage, and the absolute and variable distance measure were submitted into separate one-way ANOVAs with repeated measures for the factor condition, while Friedman tests were selected for variables that violated the assumption of normality (i.e., C-, S-, M-conditions). Next, score, save and miss percentage, and the signed distance measure were submitted to a paired *t*-test to compare performance between MLR- and MRL-conditions. The final analyses involved the same series of paired *t*-tests, but only in the trials in which participants looked to the advertisement.

## Results

Initial analysis of the Mobile-Eye data revealed that of the 16 participants, we were unable to analyse the gaze behavior of four of the participants due to issues with their video clarity. Therefore, 12 participants (all male, and right footed) were used in the final data analysis, totalling a possible number of 576 penalty kicks to be analyzed. However, a total of 564 penalty kicks were analyzed due to problems with the digital camera not having recorded every possible kick for each of the participants. To test the assumption of data normality, the Shapiro-Wilks *W*-tests were conducted on all dependent variables. In the cases that the assumption of normality was violated, Friedman and Wilcoxon Signed-Rank tests substituted parametric ANOVA'S and *t*-tests, with Dunn-Bonferonni corrections used where appropriate.

### Gaze Behavior

**Figure 3:**
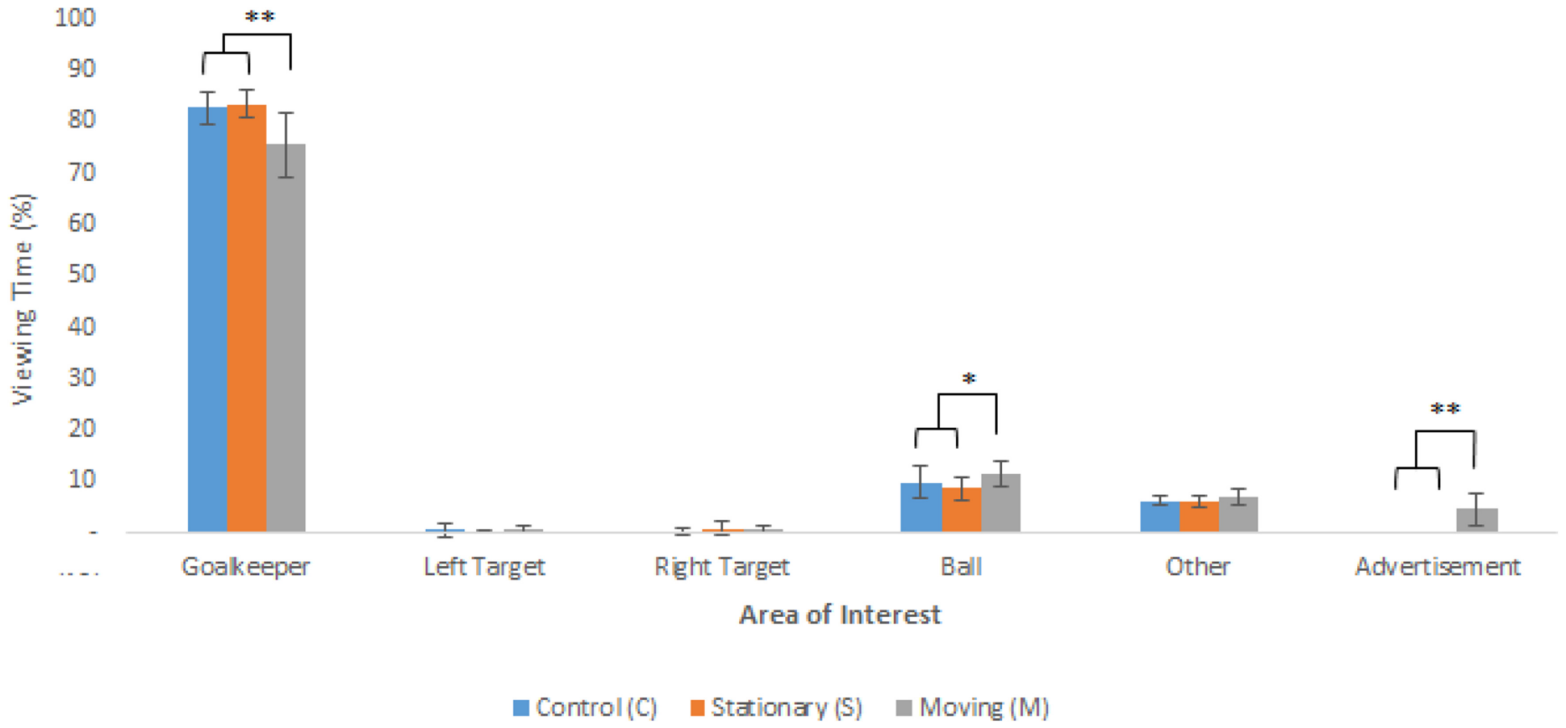
Average percentage (+SD) viewing time to each of the areas of interest between the control (C), stationary (S), and moving (M) conditions. (NB. *p < 0.05, **p < 0.001).

Figure 3 below shows the average percentage viewing time to the six areas of interest across the C-, S-, and M-conditions. In line with the instructions, the results provided a clear indication that the participants used a goalkeeper dependent strategy during the penalty kick experiment due to the high percentage of viewing time to the goalkeeper. They spent very limited time viewing the two target areas between conditions, with more, albeit brief, time spent looking to the ball and the advertisement.

The Friedman test with repeated measures results revealed a significant difference for the percentage viewing time to the goalkeeper between the three conditions [*x*^2^(3) = 10.18, *p* < 0.05]. *Post-hoc* analysis indicated that participants viewed the goalkeeper for a significantly shorter period of time in the M-condition when compared to both the C- and S-conditions. The Friedman test also showed a significant difference in percentage viewing time to the ball [*x*^2^(2) = 10.18, *p* < 0.05] with *post-hoc* analysis indicating that participants viewed the ball longer in the M-condition when compared to that of the C- and S-conditions. The percentage viewing time to other areas suggested that in the M-condition, participants viewed these locations for a longer period when compared to the other two conditions, however, the ANOVA only approached significance [*F*_(2, 22)_ = 3.22, *p* = 0.06, ηp^2^ = 0. 23]. Finally, the ANOVA for percentage viewing time to the advertisement revealed a significant main effect for condition [*F*_(2, 22)_ = 26.97, *p* < 0.001, ηp^2^ = 0.71], with *post-hoc* comparisons indicating significantly longer viewings to the advertisement in the M-condition when compared to both the C- and S-conditions.

**Figure 4:**
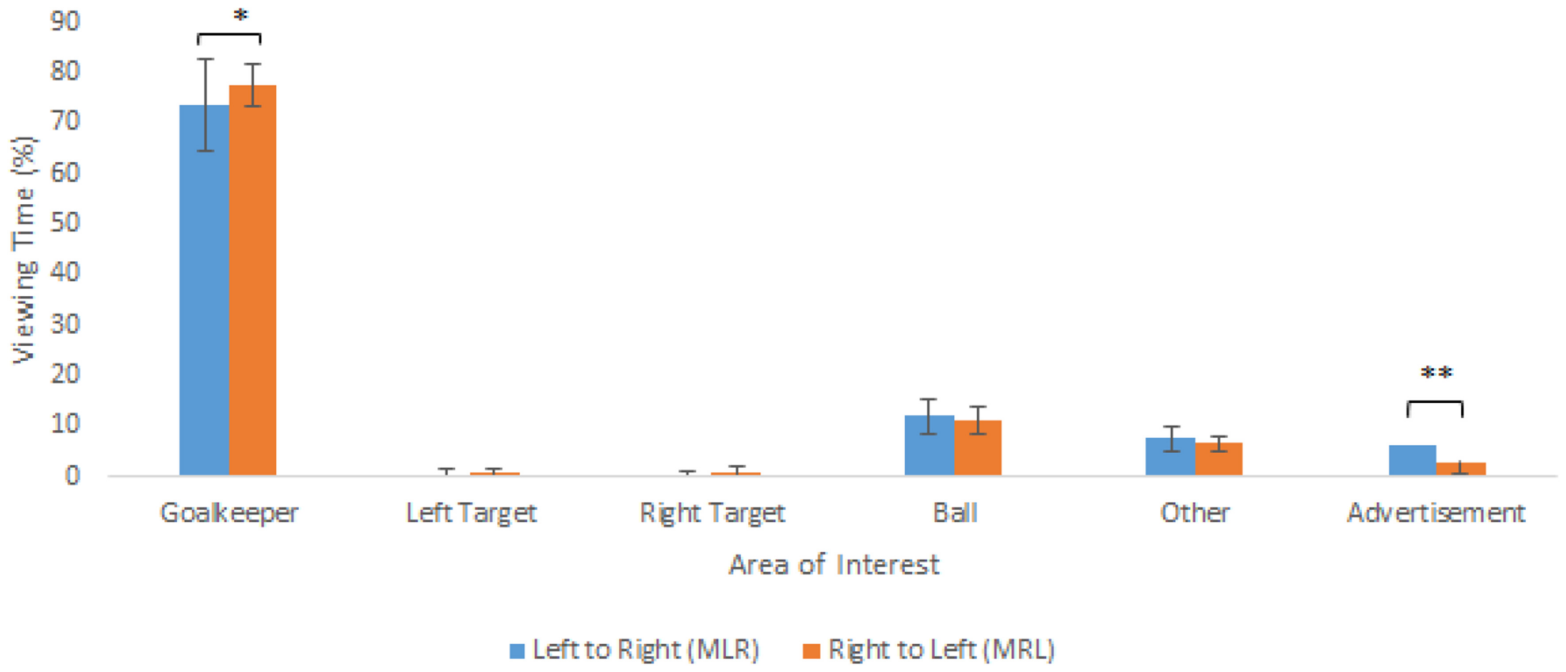
Average percentage (+SD) viewing time to the six areas of interest between the moving left to right (MLR) and right to left (MRL) conditions. (NB. *p = 0.05, **p < 0.01).

Next, we examined gaze behavior differences within the moving advertisement condition by comparing MLR- and MRL-conditions (Figure 4). Paired sampled *t*-tests indicated that the percentage viewing time was significantly less for viewing the goalkeeper [*t*_(11)_ = 2.19, *p* = 0.05, *d* = −0,63] in the MLR-condition when compared to the MRL-condition. Further to this, a paired *t*-test indicated significantly more time spent on viewing of the advertisement within the MLR-condition than in the MRL-condition [*t*_(11)_ = 3.92, *p* < 0.01, *d* = 1.13]. For the other areas of interested no significant differences between MLR- and MRL-conditions were revealed.

Finally, analyses were performed to see if there were differences in the percentage of trials that participants looked at the advertisement between MLR- and MRL-conditions. The analysis indicated that, on average, participants looked at the advertisement in 38.4% of the trials within the M-condition compared to 61.6% of trials in which they did not look at the advertisement. Further analysis showed that the participants looked to the advertisement more often in the MLR-condition than in the MRL-condition [*t*_(11)_ = 2.18, *p* = 0.05, *d* = 0.63], with the participants looking at the advertisement in 47.7% of trials in the MLR-condition and 28.6% of the trials in the MRL-condition.

### Performance Measures

With the visual search analyses indicating that participants did in fact look to the moving advertisement, we proceeded to examine the key performance measures of the penalty kick. Average score, save and miss rate as a percentage of total penalty kicks, as well as absolute and variable distance measures can be seen in Table 1. At first glance, no clear differences came to the fore.

**Table 1:** Penalty kick performance measures (Mean ± SD) between the three penalty kick conditions.

	**Control (C)**	**Stationary (S)**	**Moving (M)**
Score (%)	74.9 ± 21.4	77.7 ± 16.3	78.6 ± 14.2
Miss (%)	8.2 ± 7.8	6.3 ± 6.3	7.4 ± 7.4
Save (%)	18.0 ± 19.8	16.0 ± 16.8	14.1 ± 14.1
Opp. side GK (%)	80.6 ± 20.6	82.6 ± 17.9	84.9 ± 14.2
Absolute distance (cm)	202.0 ± 30.0	205.5 ± 33.0	205.2 ± 43.2
Variable distance (cm)	67.6 ± 24.5^*^	88.1 ± 42.7	84.6 ± 12.5^*^

* *p < 0.05*.

The separate Friedman tests found no significant difference between the number of successful penalty kicks [*x*^2^(2) = 0.17, *p* > 0.05], the percentage of missed penalty kicks [*x*^2^(2) = 0.14, *p* > 0.05], the percentage of kicks saved by the goalkeeper [*x*^2^(2) = 0.15, *p* > 0.05], or the percentage of kicks to the opposite side of the goalkeeper [*x*^2^(2) = 0.14, *p* > 0.05], between the C-, S-, and M-conditions. Similarly, Friedman tests also did not reveal significant differences for the absolute distance [*x*^2^(2) = 1.17, *p* > 0.05], yet it did show a significant difference for variable distance [*x*^2^(2) = 7.17, *p* < 0.05], with *post-hoc* analysis indicating kicks were more variable for the M-Condition when compared to the C-condition (*p* > 0.05).

Next, we examined differences within the moving condition by comparing MLR- and MRL- conditions (Table 2). A Wilcoxon Signed-Ranks test found no significant differences between the MLR- and MRL-conditions for score (Z = −0.31, *p* > 0.05), save (Z = −1.26, *p* > 0.05), miss [*t*_(11)_ = −1.60, *p* > 0.05], or kicks to the opposite side of the goalkeeper (Z = −0.99, *p* > 0.05) percentages. A paired sampled *t*-test also found no differences between the absolute [*t*_(11)_ = 0.33, *p* > 0.05, *d* = 0.10] and variable distance measures [*t*_(11)_ = −0.83, *p* > 0.05, *d* = −0.24]. However, an effect was found between the two moving advertisement conditions for the signed distance [*t*_(11)_ = −2.49, *p* < 0.03, *d* = −0.71], with the MLR-condition showing kicks that were placed to the left of the center of the goal (on average), while in the MRL-condition kicks were placed to the right of the center of the goal (on average).

**Table 2:** Penalty kick performance measures (Mean ± SD) between moving advertisement conditions.

	**Left to right (MLR)**	**Right to left (MRL)**
Score (%)	77.7 ± 18.2	79.6 ± 15.4
Miss (%)	5.6 ± 8.9	9.2 ± 7.7
Save (%)	16.7 ± 18.4	11.3 ± 12.7
Opp. side GK (%)	83.3 ± 18.4	86.6 ± 14.6
Absolute distance (cm)	207.0 ± 50.7	203.3 ± 43.4
Variable distance (cm)	81.2 ± 18.5	88.0 ± 19.0
Directional distance (cm)	−51.5 ± 87.8	22.1 ± 55.3^*^

* *p < 0.05*.

Given the fact that the results of gaze behavior showed that participants did not shift visual attention in all trials within the moving advertisement conditions, we compared performance measures between MLR- and MRL-conditions for only those trials in which they looked at the advertisement. Participant 2 was left out of the analysis as the participant did not look at the advertisement in any of the penalty kick trials in the MLR condition. This left 11 participants in the analysis (Table 3). Wilcoxon Signed-Ranks tests did not reveal significant differences between the MLR- and MRL-conditions when looking to the advertisement for score (Z = −0.84, *p* > 0.05), save (Z = −1.57, *p* > 0.05,), miss [*t*_(11)_ = −0.37, *p* > 0.05], or kicks to the opposite side of the goalkeeper (Z = −0.94, *p* > 0.05) percentages. Paired *t*-test also did not reveal significant differences for the absolute [*t*_(10)_ = 1.13, *p* > 0.05, *d* = 0.34] and variable distance measures [*t*_(10)_ = 1.15, *p* > 0.05, *d* = 0.35]. However, a significant difference was found for the signed distance measure between the MLR- and MRL-conditions [*t*_(10)_ = −2.69, *p* < 0.05, *d* = −0.81]. Results indicate that in the MLR-condition, the kicks were to the left of the center of the goal (on average), while in the MRL-condition the kicks were placed to the right side of the center of the goal (on average).

**Table 3:** Penalty kick performance measures (Mean ± SD) between moving conditions in trials to which participants looked to the advertisement only.

	**Left to right (MLR)**	**Right to left (MRL)**
Score (%)	72.9 ± 36.2	88.3 ± 16.1
Miss (%)	3.9 ± 10.2	5.3 ± 9.3
Save (%)	23.2 ± 32.5	6.4 ± 15.7
Opp. side GK (%)	76.8 ± 32.5	89.1 ± 20.2
Absolute distance (cm)	191.6 ± 87.1	161.9 ± 67.3
Variable distance (cm)	62.4 ± 38.7	42.5 ± 50.7
Directional distance (cm)	−63.3 ± 117.3	39.5 ± 101.5^*^

* *p < 0.05*.

## Discussion

With the introduction of LED billboards within competitive sport that allow moving advertisements to be displayed around soccer stadiums during game time, there is a need to understand its potential effects on visual attention. Specifically, these billboards are placed around the field, including the area behind the soccer goal area, with the potential to distract the visual attention of a player during a penalty kick, a critically decisive event within competitive soccer. It is important to understand this as previous research has demonstrated a functional coupling of gaze behavior and kicking, with the information made available from gaze fixations being pertinent in decision making, as well as maintaining effective performance (Behan and Wilson, 2008). Further to this, studies have shown the effect of distractions to visual attention, with shifts in attention to task irrelevant cues having the potential to disrupt motor performance within far-aiming tasks like the penalty kick (Beilock and Carr, 2001; Wood and Wilson, 2010; Morris, 2012; Lidor et al., 2013). We therefore investigated whether a moving advertisement positioned behind the goal area did in fact catch the visual attention of participants performing the penalty kick, and, whether this has any effects on subsequent motor performance. We hypothesized two possible effects of the moving advertisement. First, a moving background can function as a distractor, resulting in a non-specific disruption of penalty performance measures, especially in terms of aiming accuracy and precision. Alternatively, a moving background may affect the perception of target location, inducing systematic changes in penalty performance and aiming which would be specific for the direction of motion of the advertisement, analogous to the effects of the Dunker illusion observed for aiming task (e.g., Soechting et al., 2001).

It is worth noting from the initial perusal of the gaze behavior results that participants indeed used a goalkeeper dependent strategy during the experiment, with an average 78% of gaze spent looking at the goalkeeper, which was expected given the nature of the instructions to participants. The key significant finding in the gaze behavior data however was that the moving advertisement indeed caught the attention of participants compared to the no- and stationary advertisement conditions. Although gaze was affected by the motion of the background, no significant differences between the penalty performance outcome measures, namely success, miss and save rates, were found between the three conditions. Also, the participants did not significantly differ in the ability to decide and kick the ball to the opposite side of the goalkeeper dive, or differ in the accuracy of ball placement (i.e., absolute distance from the goal's center) between conditions. In fact, the only significant difference observed was with respect to the precision of ball placement (i.e., variable distance), suggesting less precise kicks in the moving advertisement condition compared to the no advertisement condition. This might suggest a small non-specific distractive effect; however, we note that the variable measure for the stationary condition was numerically (but not statistically) even higher, suggesting that the disruptive effect, if any, is not induced by the motion of the advertisement.

When comparing gaze behavior between the moving advertisement conditions, we found that participants' visual attention shifted in more trials to the advertisement when it moved from left to right compared to when moving right to left. Presumably, this also lead participants, on average, to spend more time viewing the advertisement when it moved from left to right. In relation to this finding, it has been found that when looking at pictures of natural scenes, neurologically intact individuals show a leftward bias in the direction of their first eye movement. The presence of this leftward bias within spatial attention is known as pseudoneglect (Nuthmann and Matthias, 2014; Hartmann et al., 2019; see Nicholls et al., 2010; Noël et al., 2015 for pseudoneglect in kicking tasks). This as well as the fact that the angle of the run up for the right footed players place the advertisement in the corner of the eye, could be the reason why the participants were more likely to look to the advertisement within the left to right condition. We did only include right-footed players within the present study. Future research should consider whether similar findings occur within left-footed players due to the differing constraints on the run-up for these players, and also because some authors have argued that pseudoneglect effects may be lateralized (McCourt and Garlinghouse, 2000).

Although visual attention differed, we found no measurable difference in the penalty performance outcome measures between the two moving advertisement conditions. Yet, when aiming is concerned, a significant effect on the ball landing location was found: kicks were aimed to the left of the center of the goal when the advertisement moved from the left to right, while kicks were placed to the right of the center of the goal when the advertisement moved in the opposite direction. This theoretically pertinent result is aligned with previous findings in pointing and hitting using the Duncker illusion (Soechting et al., 2001; Brouwer et al., 2003), in which the presence of background motion from left to right and right to left resulted in systematic aiming errors to the left and right of a target, respectively. Taking this as well as later findings that accuracy of far aiming tasks is impacted by allocentric or contextual information (Van der Kamp and Masters, 2008; Van der Kamp et al., 2009; Shim et al., 2014), our results suggest the exploitation of allocentric information sources in far-aiming task like the penalty kicks. Initially, this would seem in contradiction to the two visual systems model proposed by Milner and Goodale (2008), as the control of action typically exploits egocentric information. Yet, as Van der Kamp and Masters (2008), Van der Kamp et al. (2009), Shim et al. (2014) have suggested, aiming tasks such as the penalty kick may involve identifying landing location and this process may actually be more consciously controlled and thus involve the ventral stream, thereby exploiting allocentric information (see also Willingham, 1998). To what degree, the, presumably unintended, systematic effects on aiming accuracy can actually also bring about a degradation in performance outcome with a real goalkeeper trying to save the penalty kick must be addressed in future studies. That is, in the present study, the exact aiming location did not affect the performance outcome scores, but when using a real goalkeeper, differences in aiming can potentially bring the ball (just) within reach, affecting the opportunities for the goalkeeper to intercept the ball.

A few additional notes have to be made regarding the findings that relate to representativeness of the current experimental procedures. With our current design, the situation enforced a keeper-dependent strategy which has allowed us to maximize the penalty takers visual attention to the goalkeeper and the goal area. However, it is important seek to which degree these findings can be generalized to kickers who use a goalkeeper-independent strategy. Due to the differences in visual gaze patterns across the two penalty taking strategies, it is pertinent to understand the effects on visual attention and subsequent penalty performance within the goalkeeper-independent strategy as well. A likely difference is toward the timing of the effect. While with the current keeper-dependent strategy, a moving advertisement can affect aiming almost through the entire run-up and kick, it is likely that with a goalkeeper-independent strategy the effect is restricted to the preparation and early phase of the run-up, because within the keeper-independent strategy, kickers tend to focus their attention earlier and longer toward the ball. Also, the length of the run-up is a potential factor influencing the relative amount of time kickers spend looking at the goalkeeper, goal and ball, and thus, their susceptibility to a moving advertisement (Kurz et al., 2018). Another concern might be that in the competitive environment, kickers only have one attempt to complete a penalty kick and previous research has suggested that participants tend to adjust penalty strategies as the trials continue in order to be more successful (Wood and Wilson, 2010), and in the current study also may have adapted to the attention drawing effect of the moving advertisement, having less effect over time. It would be interesting to see the effects of a moving advertisement in a single attempt in future studies in order to better mimic the competitive situation. We do think, however, that with respect to the information available for aiming and the spatial constraints on action our design is reasonably representative relative to on-field or competitive situations. In fact, the major flaw in terms of representative design is in the absence of dynamic interactions between kicker and goalkeeper. This relates to kicker being instructed to use a keeper-dependent strategy (as discusses above) and the use of a goalkeeper projection, which -obviously- did not respond to the kickers' action. Importantly, therefore, future research must verify the observed effects of moving advertisement in on-field environments, for instance, by analyzing video-footage of competitions. A final but relevant concern would be the difference between the pressure perceived by the participants during the current study, vs. the pressure experienced in a competitive environment. Attentional control theory (ACT) propose that anxious individuals both orient more rapidly to salient or conspicuous stimuli, and disengage from them more slowly (Wilson et al., 2009). This is theoretically interesting as implications are that in higher anxiety competitive situations, a moving advertisement could affect attention even more that in the penalty kickers shown within this study. Future research, including notational analysis of video-footage, should take this into consideration.

In conclusion, a moving advertisement placed behind the goal area was found to catch the visual attention of soccer penalty kickers using a goalkeeper-dependent strategy, with no measurable distractive non-specific effects on penalty kick performance measures. However, importantly, systematic effects on aiming were found when comparing conditions in which the advertisement moved in opposite directions suggesting that the aiming accuracy of the penalty kick is impacted by task-irrelevant contextual information.

## Data Availability Statement

The datasets generated for this study are available on request to the corresponding author.

## Ethics Statement

The studies involving human participants were reviewed and approved by Scientific and Ethical Review Committee, Faculty Committees, Faculty of Behavioral and Movement Sciences. The patients/participants provided their written informed consent to participate in this study.

## Author Contributions

Article was planned by GP, JK, and GS. Data was collected by GP. Data analyzed by GP and JK. Article write up by GP, assisted by JK, and final sign off by GS.

### Conflict of Interest

The authors declare that the research was conducted in the absence of any commercial or financial relationships that could be construed as a potential conflict of interest.

## Footnotes

^1^The choice to encourage the participants to use of a keeper-dependent strategy was to ensure that during every penalty kick, participants would maximize (visual) attention toward the goalkeeper and goal while running up to and kicking the ball. That is, with a keeper-independent strategy, kickers typically focus earlier and longer exclusively to the ball (and perhaps even more so within the repetitive circumstance of an experiment).
